# A/T/(N) Profile in Cerebrospinal Fluid of Parkinson’s Disease with/without Cognitive Impairment and Dementia with Lewy Bodies

**DOI:** 10.3390/diagnostics10121015

**Published:** 2020-11-26

**Authors:** Giovanni Bellomo, Federico Paolini Paoletti, Elena Chipi, Maya Petricciuolo, Simone Simoni, Nicola Tambasco, Lucilla Parnetti

**Affiliations:** 1 Laboratory of Clinical Neurochemistry, Section of Neurology, Department of Medicine, University of Perugia, 06132 Perugia (PG), Italy; giovanni.bellomo@unipg.it (G.B.); mayapetricciuolo@gmail.com (M.P.); 2 Section of Neurology, Department of Medicine, University of Perugia, 06132 Perugia (PG), Italy; federico.paolinipaoletti@gmail.com (F.P.P.); elena.chipi@outlook.com (E.C.); simonesimoni84@gmail.com (S.S.); n.tambasco@libero.it (N.T.)

**Keywords:** parkinson’s disease, dementia with lewy bodies, mild cognitive impairment, CSF biomarkers

## Abstract

Neuropathological investigations report that in synucleinopathies with dementia, namely Parkinson’s disease (PD) with dementia (PDD) and dementia with Lewy bodies (DLB), the histopathological hallmarks of Alzheimer’s Disease (AD), in particular amyloid plaques, are frequently observed. In this study, we investigated the cerebrospinal fluid (CSF) AD biomarkers in different clinical phenotypes of synucleinopathies. CSF Aβ42/Aβ40 ratio, phosphorylated tau and total tau were measured as markers of amyloidosis (A), tauopathy (T) and neurodegeneration (N) respectively, in 98 PD (48 with mild cognitive impairment, PD-MCI; 50 cognitively unimpaired, PD-nMCI), 14 PDD and 15 DLB patients, and 48 neurological controls (CTRL). In our study, CSF AD biomarkers did not significantly differ between CTRL, PD-MCI and PD-nMCI patients. In PD-nMCI and PD-MCI groups, A-/T-/N- profile was the most represented. Prevalence of A+ was similar in PD-nMCI and PD-MCI (10% and 13%, respectively), being higher in PDD (64%) and in DLB (73%). DLB showed the lowest values of Aβ42/Aβ40 ratio. Higher total tau at baseline predicted a worse neuropsychological outcome after one year in PD-MCI. A+/T+, i.e., AD-like CSF profile, was most frequent in the DLB group (40% vs. 29% in PDD).

## 1. Introduction

Parkinson’s disease (PD) is the most common neurodegenerative disease with motor disturbances among the elderly [1]. The clinical presentation includes the core motor signs (bradykinesia, rigidity and resting tremor) as well as non-motor symptoms [2,3]. Among the latter, cognitive impairment is one of the most disabling, with a deleterious impact on the quality of daily life. Almost 30% of PD patients are affected by mild cognitive impairment (PD-MCI), which in turn represents a risk factor for the subsequent development of PD with dementia (PDD) [4]. The frequency of PD-MCI and PDD typically increases with disease duration. However, cognitive deficits can also appear at the earliest stages of PD [5]. Cognitive impairment and parkinsonism coexist in other neurodegenerative disorders. Among them, dementia with Lewy bodies (DLB) shares with PD the presence of neuronal proteinaceous inclusions like Lewy bodies (LB) and Lewy neurites [6]. The neuropathological hallmarks of Alzheimer’s Disease (AD), in particular amyloid plaques, are also reported in synucleinopathies [7,8]. Post-mortem studies showed that the central nervous system (CNS) amyloid burden can be even higher in DLB with respect to AD and that it correlates with the development of dementia better than α-synuclein pathology both in PDD and DLB [7,8,9]. Whereas neuropathological evidences provide the final picture of a neurodegenerative disease, cerebrospinal fluid (CSF) biomarkers are able to track the pathological molecular processes along the disease course in vivo. Previous studies found lower CSF Aβ42 and higher total tau (t-tau) concentrations in DLB patients compared to PDD and PD patients [10,11,12]. CSF Aβ42 provided a robust prognostic value in terms of cognitive decline both in PD and DLB [13,14,15]. Nevertheless, the impact of AD-related pathology on the different cognitive manifestations of PD (PD, PD-MCI, PDD) has not been completely elucidated.

The National Institute on Aging and Alzheimer’s Association (NIA-AA) research framework recently proposed a classification model based on the categorical classification of biomarkers, namely the A/T/(N) system. In this model, “A” refers to amyloidosis, “T” refers to tauopathy and “N” refers to neurodegeneration [16,17]. According to this model, AD is defined by biomarkers evidence of cerebral amyloidosis (A+) and tauopathy (T+), with neurodegeneration (N+), usually reported in round brackets, being not a necessary condition. Since the coexistence of AD-related pathology may participate in the cognitive decline in other protein misfolding diseases, the use of the A/T/(N) system could be useful in detecting an underlying AD pathology in synucleinopathies [18].

In this study, we investigated, through CSF biomarkers, the presence of AD fingerprints in different clinical phenotypes of synucleinopathies. We measured CSF Aβ42/Aβ40 ratio, tau protein phosphorylated at threonine 181 (p-tau) and t-tau as markers of amyloidosis (A), tauopathy (T) and neurodegeneration (N) respectively, in a cohort of PD (cognitively unimpaired, PD-MCI and PDD), DLB patients and neurological controls (CTRL).

## 2. Materials and Methods

### 2.1. Patients

A total number of 127 patients and 48 control subjects (CTRL) were considered in the present study. Patients with PD, PDD and DLB were consecutively recruited among patients referring to the Movement Disorders Centre and Centre for Memory Disturbances of our Neurology Section from 2016 to 2019. Clinical diagnosis of PD, PDD and DLB was made according to the currently available criteria [2,19]. In particular, PD and DLB patients satisfied the diagnostic criteria for clinically established PD [2] and probable DLB [19], respectively. All the patients underwent a baseline visit consisting of comprehensive neurological evaluation and neuropsychological assessment. Patients with PDD and DLB completed the Montreal Cognitive Assessment (MoCA) [20] and the Mini-Mental State Examination (MMSE) [21] as a measure of global cognitive functioning. In addition to MoCA and MMSE, PD patients completed an extensive neuropsychological battery (Table S1, Supplementary Materials) comprehensive of two tests within each of the five domains (attention and working memory, executive, language, memory and visuo-spatial), according to the Movement Disorder Society (MDS) recommendations criteria [22]. Accordingly, patients were classified as having MCI based on MDS recommendations for PD-MCI level II criteria, requiring an impaired performance (more than 1.5 standard deviations below the mean) on at least two standard neuropsychological tests in one domain or in different cognitive domains.

In PD patients, clinical motor impairment was rated according to the examination of Movement Disorders Society Unified Parkinson’s Disease Rating Scale III (UPDRS-III) [23]. Also, disease-related disability was assessed by means of the Hohen and Yahr staging scale (H&Y) [24]. All the patients underwent brain imaging (Magnetic Resonance Imaging and/or Computed Tomography) and lumbar puncture (LP). CSF was collected according to the hospital standard protocol. For all the patients, disease duration was calculated in months from the onset of the first symptoms to the time of LP. 60 PD and 4 PDD patients underwent motor or neuropsychological assessment one year after LP. Follow-up visits included neuropsychological evaluations (MMSE and MoCA) and motor assessment. Among them, 2 PDD, 21 PD-MCI patients and 25 PD-nMCI patients completed MMSE and MoCA, both at baseline and at follow-up.

The CTRL group was composed of cognitively unimpaired subjects, who underwent lumbar puncture for subjective memory complaints not confirmed by the neuropsychological assessment, or as part of a diagnostic work-up for minor neurological symptoms (i.e., headache, peripheral neuropathy, etc.), showing no cognitive impairment after at least a 2-year follow-up. The study was approved by the local Ethical Committee (ID code: marcatori liquorali nelle malattie neurodegenerative, protocol N°: 19369/08/AV, registry N°: 1287/08, date: 9 October 2008).

### 2.2. CSF Sampling and Analysis

LP was performed according to international guidelines [25]. Following a standardized procedure, 10–12 mL of CSF was collected in sterile polypropylene tubes and centrifuged at room temperature for 10 min (2000× *g*). Aliquots (0.5 mL) were frozen at −80 °C. In the CSF samples collected before 2018, Aβ40 and Aβ42 levels were measured by using the EUROIMMUN enzyme-linked immunosorbent assay (ELISA) kit (EUROIMMUN AG, Lübeck, Germany), while t-tau and p-tau levels were measured by using INNOTEST ELISA (Fujirebio Europe, Gent, Belgium). In the CSF samples collected since 2018, the assays were performed using Lumipulse G600-II (Lumipulse) β-Amyloid 1–40, Lumipulse β-Amyloid 1–42, Lumipulse Total Tau and Lumipulse p-Tau 181 assays (Fujirebio Europe, Gent, Belgium). Different production lots numbers were used to generate data from both EUROIMMUN and INNOTEST manual ELISAs and for all Lumipulse assays. Standard quality controls and internal quality controls were assayed in each run. The number of samples analyzed with Lumipulse and ELISAs is reported for each biomarker and for each diagnostic group in Supplementary Tables S2 and S3. The A/T/(N) profile was considered for each patient. A/T/(N)-related biomarkers’ values were classified as pathological (+) or not pathological (−) by using two sets of slightly different cut-off values depending on the assay used for the analysis. For the samples analyzed using manual ELISA methods, cut-offs were 0.076, 64.5 and 400 pg/mL, for Aβ42/Aβ40 ratio, p-tau and t-tau, respectively. For the samples analyzed using Lumipulse assays, cut-offs were 0.069, 56.5 and 404 pg/mL, for Aβ42/Aβ40 ratio, p-tau and t-tau, respectively. Patients with both pathologic Aβ42/Aβ40 ratio (under the cut-off value) and pathologic p-tau (above the cut-off value) were considered as having a CSF AD-like profile (A+/T+). Neurodegeneration (N+) was defined for t-tau values above the cut-off value.

### 2.3. Statistical Analysis

The data analysis was performed by using OriginPro 9 and R v3.6. Demographical and clinical features were summarized by using the mean and its 95% confidence interval (CI). The significance of unequal male/female percentages in groups was assessed by the deviance from a binomial distribution with a male/female ratio of 1. Differences in continuous demographical and clinical variables among groups were tested by one-way analysis of variance (ANOVA), using least significant difference (LSD) as a post-hoc test. The significance of differences in count data among groups were assessed by a generalized version of Fisher’s Exact test [26]. The significance of the different prevalences of A+, T+ and N+ between groups was assessed by binomial logistic regression by considering age as a covariate variable. The significance of differences in continuous biomarker levels among groups were tested by two-way analysis of covariance (ANCOVA) by assuming age as a covariate variable. Since for CTRL and PD-nMCI groups t-tau could not be assumed to be normally distributed by Kolmogorov–Smirnov normality test, a non-parametric version of the ANCOVA analysis was also performed (with results very similar to the parametric version) and it is reported in the Supplementary Materials (Table S4). Correlations between CSF biomarkers and baseline clinical parameters (MoCA, MMSE, UPDRS-III and H&Y) were calculated on the PD-nMCI and PD-MCI groups separately by means of Spearman’s (ρ) correlation coefficient, since normality could not be assumed for all the tested biomarkers (t-tau in CTRL and PD-nMCI groups) by applying the Kolmogorov–Smirnov normality test. For each test used, a *p*-value below 0.05 was considered significant to reject the null hypothesis. *p*-values smaller than 0.001 were represented as *p* < 0.001. All the *p*-values calculated in the correlation analysis were corrected for multiple testing according to Benjamini and Hochberg [27].

## 3. Results

Our study cohort was composed as follows: 98 PD patients, 14 PDD patients, 15 DLB patients and 48 neurological controls (CTRL). Among PD patients, 48 satisfied MCI criteria (PD-MCI), while the remaining PD patients (*n* = 50) were cognitively unimpaired (PD-nMCI). 69 PD patients were de novo, whereas 29 were on treatment: de novo PD patients came to our attention for the first time for disease characterization, and all of them were drug-naïve, independent of clinical stage. Patients on treatment (PD-T) showed a mean value of Levodopa Equivalent Daily Dose (LEDD) of 610 mg (standard deviation = 404 mg). Demographical features, clinical features and dopaminergic treatments of each of these groups are summarized in Table 1. Of importance, the CTRL, PD-nMCI, PD-MCI, PDD and DLB groups showed significant differences in age, as assessed by one-way analysis of variance (ANOVA) (*p* < 0.001). In particular, PD-nMCI patients were significantly younger than PD-MCI (*p* < 0.001), PDD (*p* < 0.001) and DLB patients (*p* < 0.001), while CTRL subjects were significantly younger with respect to DLB (*p* = 0.01) and older with respect to PD-nMCI (*p* = 0.015). A higher number of males than females was observed among DLB, PDD and PD patients, with a significant deviance from a 50% binomial distribution (*p* < 0.001) and from the CTRL group (*p* = 0.009). Both H&Y and UPDRS-III did not significantly differ among PD-nMCI, PD-MCI and PDD groups, although the differences in H&Y scores between PDD and PD-nMCI were towards significance. Cognitive scores (MoCA and MMSE) significantly differed among groups with dementia (PDD and PDD), PD-MCI and cognitively unimpaired subjects (PD-nMCI and CTRL). The fraction of treated patients for each type of treatment prescribed did not significantly differ between PD-nMCI and PD-MCI groups.

**Figure 1:**
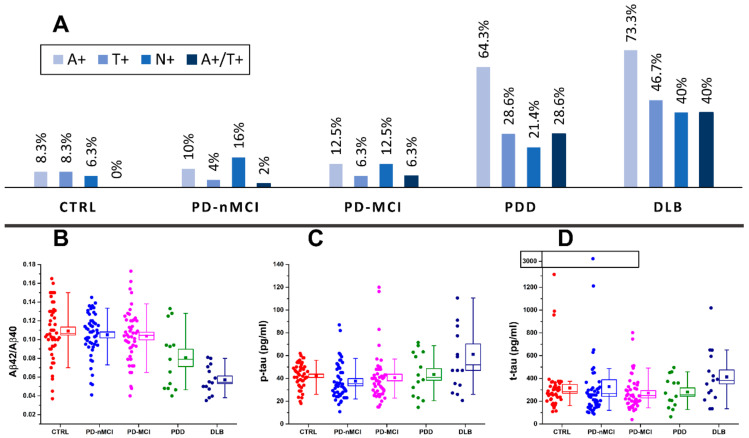
(**A**) prevalence of amyloidosis (A+), tauopathy (T+), neurodegeneration (N+) and A+/T+ in cognitively unimpaired PD (PD-nMCI), PD patients with mild cognitive impairment (PD-MCI), PD with dementia (PDD) and dementia with Lewy bodies (DLB) patients and control (CTRL) subjects. Distribution of amyloid-β (Aβ)42/Aβ40 (**B**), phosphorylated tau (p-tau) (**C**) and total tau (t-tau) (**D**) biomarker values across CTRL, PD-nMCI, PD-MCI, PDD and DLB groups. Boxes representing data distributions are centered on the mean values, with the internal horizontal line representing the median. Box heights are equal to the standard error of mean values while whiskers represent the 10–90% data range.

The frequencies of A+, T+, N+, A+/T+ and the different values of Aβ42/Aβ40, p-tau and t-tau across the different diagnostic groups are shown in Figure 1. Considering the significant age differences among the diagnostic groups and the known correlations between biomarker values and age, both the differences in continuous biomarker values and in A+, T+, N+ and A+/T+ frequencies were assessed pairwise between diagnostic groups by controlling for age. The *p*-values of this analysis are displayed in Table 2. With respect to continuous biomarkers values, the fraction of samples analyzed with ELISA and Lumipulse was similar among all the diagnostic groups (see Table S2, Supplementary Materials). The number of samples analyzed with different techniques were comparable, as shown by Fisher’s exact test for count data on group pairs for each biomarker. By considering the most represented pathology (PD), we found no significant differences between mean values for Aβ42/Aβ40 and p-tau (approximately normally distributed) and between medians for t-tau (not normally distributed) in samples measured with different assays (see Table S3, Figures S1–S3 in the Supplementary Materials).

With respect to Figure 1, the Aβ42/Aβ40 (A+) ratio was pathologically reduced in the majority of DLB patients (73%), in more than half of PDD cases (64%), in 10% of PD-nMCI patients, 13% in PD-MCI patients and in 8.3% of CTRL subjects. By looking at the *p*-values reported in Table 2, both Aβ42/40 values and A+ prevalence were significantly different in DLB and PDD groups compared to CTRL, PD-nMCI and PD-MCI. CSF p-tau was pathologically increased (T+) in more than a third of DLB patients (47%) and in more than a quarter of PDD cases (29%), whereas it was normal in almost all CTRL subjects (8.3%) and in PD-nMCI and PD-MCI patients (4% and 6% of prevalence, respectively). Both p-tau concentration and T+ prevalence in DLB were significantly higher as compared to CTRL, PD-nMCI and PD-MCI. In PDD, p-tau levels were not significantly higher with respect to CTRL, PD-nMCI and PD-MCI, whereas the higher prevalence of T+ was toward significance. CSF t-tau concentrations were significantly different only for DLB vs. PD-MCI. However, t-tau was pathologically increased (N+) in 40% of DLB patients, in 21% of PDD patients, in 6.3% of CTRL subjects, in 16% of PD-nMCI patients and in 14% of PD-MCI patients. The prevalence of patients exhibiting N+ showed a trend towards higher values in DLB and PDD with respect to CTRL, PD-nMCI and PD-MCI but, while adjusting for age differences, it reached the statistical significance only for DLB vs. CTRL. Four of the 14 patients with PDD (29%) and 6 of the 15 DLB (40%) patients had a CSF AD-like profile (A+/T+) with a significantly higher prevalence with respect to PD-MCI and PD-nMCI.

By considering the three assessed biomarkers, PD-nMCI, PD-MCI and CTRL groups showed substantially overlapping CSF biomarkers and A/T/(N) profiles with a prevalence of A-/T-/N-. No statistically significant differences were found pairwise among these groups (Table 2) nor by considering multiple groups comparison tests (age-corrected ANOVA for biomarker values and Fisher’s exact test for A+, T+ and N+ prevalence).

Correlations between biomarkers and clinical and neuropsychological scores were evaluated separately in PD-nMCI and PD-MCI groups. After adjusting *p*-values for multiple testing, only few correlations were statistically significant. Within the PD-nMCI group, MoCA scores at baseline negatively correlated with t-tau, with ρ = –0.49 (*p* = 0.003).

At one-year follow-up, there were slight differences in MoCA, MMSE, UPDRS-II and H&Y scores (see Table 3). The mean values of these differences (Δ) were all null within their 95% CI and not significantly different between PD-nMCI and PD-MCI.

In the PD-MCI group, with respect to differences in clinical and neuropsychological scores at follow-up, the worsening of MMSE scores significantly correlated with t-tau, with ρ = –0.62 (*p* = 0.012). No significant correlations between biomarkers and clinical scores (UPDRS-III and H&Y) were found, nor at baseline or considering changes at follow-up.

## 4. Discussion

CSF biomarkers are of substantial help in detecting the underlying pathological substrates of CNS neurodegenerative disorders in vivo. This holds true for the role of cortical amyloid burden in determining cognitive impairment and/or representing a negative outcome for cognitive impairment in different conditions, including synucleinopathies [10,14,28,29]. In this respect, CSF Aβ42/Aβ40 ratio is nowadays considered the most reliable indicator of brain amyloid burden [30]. Neuropathological studies show that DLB represents the synucleinopathy characterized by the most prominent brain amyloidosis, frequently showing a concomitant Alzheimer’s pathology [31]. A recent study investigated CSF AD biomarkers in a large multicenter cohort including PD patients with and without dementia and DLB patients. It revealed a CSF AD profile with a hierarchy DLB > PDD > PD [11]. Besides PDD and DLB, we investigated CSF AD biomarkers in a well-characterized cohort of PD patients, distinguishing cognitively unimpaired PD and PD-MCI patients. As main results, we found that PD-MCI patients did not differ from a group of cognitively unimpaired PD patients and from CTRL subjects for any of the investigated biomarkers, and that the AD CSF profile was most frequently observed in DLB.

The A-/T-/N- profile was similarly prevalent in CTRL, PD-nMCI and in PD-MCI, with very low frequencies of A+ (8.3%, 10% and 13%, respectively) and T+ (8.3%, 4% and 6%, respectively). These results suggest that MCI in PD is not invariably linked to the presence of amyloid plaques and/or neurofibrillary tangles, rather it could result from the interplay of other pathological mechanisms, including those related to α-synuclein pathology. 

So far, contradictory results have emerged from previous studies assessing CSF AD biomarkers in PD patients with and without MCI [32,33,34,35,36]. This inconsistency among our and previous studies might be due to the heterogeneity of study cohorts (i.e., early untreated patients vs. treated patients with more advanced disease stage, different neuropsychological instruments, unselected cases vs. patients referring to expert specialized centers). Consistently with our results, two independent clinical-pathological studies on PD-MCI previously reported a prominent representation of Lewy bodies (LB)-related pathology, with only a small portion of PD-MCI with amnestic pattern showing diffuse amyloid and neuritic plaques in the cerebral cortex [37,38]. In our cohort, the longitudinal analyses showed slight differences (null within their 95% CI) between the MMSE and MoCA at 1-year follow-up. PD-nMCI showed improvement in both scales’ mean scores, which might be consistent with fluctuations of cognition or practice effect [39]. Instead, PD-MCI showed a slight decline in MMSE. In line with previous findings [40], these results suggest that MMSE may be better for tracking cognitive decline in PD-MCI, compared to MoCA, which in turn may be more sensitive in detecting early cognitive deficits.

Considering the relation between biomarkers and neuropsychological scores, in PD-MCI patients, baseline CSF t-tau was associated to a decrease of MMSE scores after 1-year follow-up (ρ = –0.62). Accordingly, a significant association between t-tau and the rate of cognitive decline was already reported [41,42]. We did not find any significant association between Aβ42/Aβ40 ratio and cognitive decline, partly due to the small number of patients and the short follow-up. 

AD-like profile (A+/T+) was most frequently found in DLB (40%) and PDD (29%). In a previous single-center report [10], 21% of DLB and 11% of PDD revealed an AD-like CSF pattern. A subsequent large multicenter study showed a CSF AD-like profile in 25% of DLB patients, 9% of PDD patients and 3% of PD patients [11]. In our cohort, the higher prevalence of A+/T+ CSF profile probably reflects the use of the Aβ42/Aβ40 ratio, which is a more sensitive and accurate marker of brain amyloidosis with respect to the sole Aβ42 [30]. Clinical and demographical features of heterogeneous cohorts contribute to explain these differences in terms of AD prevalence among distinct studies. DLB and PDD showed a more pronounced amyloid burden compared to PD considering the differences both in biomarker values (CSF Aβ42/Aβ40 ratio in DLB < PDD and in PDD < PD) and in A+ frequencies (DLB and PDD > PD, with the highest prevalence in DLB). Our results remain consistent with post-mortem studies describing an amyloid burden hierarchy DLB > PDD > PD [43,44,45] and with in-vivo studies revealing lower CSF levels of Aβ42 in DLB vs. PDD and in DLB and PDD vs. PD [11,12,46]. In our cohort, T+ prevalence was higher in DLB compared to PD. Accordingly, higher CSF p-tau levels were more frequently found in DLB than in the other groups (DLB > PDD > PD ~ CTRL). Neuropathological studies show that in DLB neurofibrillary tangles are poorly represented and confined to allocortical areas [31,47]. Recent evidences suggest that neurofibrillary tangles burden in synucleinopathies is a strong predictor of worse outcome in both motor and cognitive domains [48]. In our cohort, A+ positivity was found in 8.3% of CTRL. This result, consistent with previous data [49,50,51,52], confirms that amyloidosis can also be observed in cognitively normal individuals. Studies assessing amyloid pathology in cognitively unimpaired individuals reported prevalence of amyloid pathology, by using CSF or positron emission tomography (PET), ranging between 10% and 70% [51]. According only to CSF analysis, decrease in Aβ 42 levels or Aβ42/40 ratio was found in 21% of individuals, as reported in a recent meta-analysis [49].

As limitations of our study, we must acknowledge the relative paucity of patients in the PDD and DLB groups and the short follow-up of PD patients. A longer follow-up is needed to evaluate conversion and reversion rates and to identify further correlations between CSF biomarkers and neuropsychological scores. The use of two different techniques for biomarkers measurement may also represent an issue. However, the use of differential cut-off values prevented inconsistencies in the analysis of A+, T+, N+ and A+/T+ prevalence. Furthermore, no significant differences were found in terms of fractions of samples tested with ELISA/Lumipulse among groups nor in the biomarker concentrations measured with the different assays within the most populated diagnostic group (the PD group).

In conclusion, we measured CSF core AD biomarkers in different clinical phenotypes of synucleinopathies, ranging from cognitively unimpaired PD to PD with MCI, dementia and DLB. Differently from previous investigations, as a more reliable measure of brain amyloidosis, the Aβ42/Aβ40 ratio was used instead of Aβ42 alone, and the CSF A/T/(N) profile was considered for each group. As compared to continuous CSF biomarkers’ values, the use of CSF A/T/(N) profile provides a clear dichotomous response in terms of amyloidosis, tauopathy and neurodegeneration. In our analysis, CSF AD biomarkers showed different patterns in synucleinopathies, being most prevalent in DLB. Differently from DLB and PDD, our data suggest that PD-MCI does not seem to be invariably linked to AD biomarkers. Thus, it is most probable that MCI in PD represents a highly heterogeneous entity not directly linked to Aβ or tau misfolding [53]. Further studies with larger cohorts and longer follow-up will help to clarify this issue.

## Figures and Tables

**Table 1:** Demographical and clinical features of the study cohort. Age, education (edu.), disease duration (d. d.), Unified Parkinson’s Disease Rating Scale III (UPDRS-III), Hohen and Yahr staging scale, Montreal Cognitive Assessment (MoCA), Mini-Mental State Examination (MMSE) and Levodopa Equivalent Daily Dose (LEDD) values among groups are reported as mean (95% confidence interval - CI). Multiple groups comparison was performed by one-way analysis of variance (ANOVA) for age, edu., dd., UPDRS-III, H&Y, MoCA, MMSE and LEDD. Multiple groups comparison on count data was performed by Fisher’s exact test, i.e., for males/females (M/F), number (n.) of treated Parkinson’s disease (PD) patients (PD-T), n. of PD-T treated with levodopa (L-dopa), n. of PD-T treated with dopamine agonists (DA), n. of PD-T with Monoamine oxidase inhibitors (MAO-B-I) and n. of PD-T with catechol-o-methyltransferase inhibitors (COMT-I).

	PD-nMCI	PD-MCI	PDD	DLB	CTRL	Multiple Groups Comparison *p*-Values
*n*	50	48	14	15	48	
**Age (y)**	61 (59, 64)	67 (66, 69)	72 (66, 77)	73 (69, 77)	66 (63, 69)	<0.001
**M/F**	32/18	36/12	12/2	12/3	22/26	0.009, only CTRL differs
**Edu. (y)**	11.7 (10.6, 12.7)	9.6 (8.5, 10.8)	8.4 (5.6, 11.2)	7.2 (5.3, 9.1)	11.0 (9.6, 12.3)	<0.001, lower in DLB
**d. d. (mo.)**	32 (22, 43)	28 (18, 37)	74 (46, 101)	34 (22, 45)	-	<0.001, longer d. d. in PDD
**UPDRS-III**	26.0 (22.7, 29.3)	29.3 (26.9, 31.8)	27.9 (20.1, 35.6)	-	-	0.31
**H&Y**	1.98 (1.82, 2.14)	2.23 (2.13, 2.33)	2.14 (1.59, 2.69)	-	-	0.08
**MoCA**	25.4 (24.4, 26.3)	19.4 (18.2, 20.7)	13.1 (9.8, 16.3)	15 (11.4, 18.6)	-	<0.001
**MMSE**	28.5 (28.0, 28.9)	26.2 (25.6, 26.8)	18.9 (15.7, 22.1)	20 (15.6, 24.4)	28.3 (28.0, 28.7)	<0.001
**PD-T (n.)**	15	14	13	-	-	<0.001, only PDD differs
**L-dopa (n.)**	12	13	12	-	-	<0.001, only PDD differs
**DA (n.)**	11	10	3	-	-	0.99
**MAO-B-I (n.)**	6	4	3	-	-	0.36
**COMT-I (n.)**	1	1	4	-	-	<0.002, only PDD differs
**LEDD (mg)**	696 (404, 988)	518 (409, 626)	518 (399, 716)	-	-	0.39

**Table 2:** Age-adjusted *p*-values relative to biomarker differences between different diagnostic groups are represented in a matrix-like fashion. The *p*-values inserted in the lower diagonal part of the matrices (green cells) are relative to continuous markers (Aβ42/Aβ40, p-tau and t-tau), while the *p*-values inserted in the upper diagonal part are relative to A/T/(N) prevalence (magenta cells). Differences in continuous markers between diagnostic groups were assessed by two-way analysis of covariance (ANCOVA) by controlling for age. Differences in A/T/(N) binary outcomes between diagnostic groups were assessed by binomial logistic regression by controlling for age. Differences in A+/T+ prevalence were not assessed for CTRL since the category was not represented (by definition) in the group. Cells relative to significant *p*-values (<0.05) are represented in bold.

Continuous Marker			A/T/(N)		
Aβ42/Aβ40 (A+) ~ age + group					
	CTRL	PD-nMCI	PD-MCI	PDD	DLB
CTRL	-	0.42	0.44	**<0.001**	**<0.001**
PD-nMCI	0.16	-	0.81	**0.003**	**0.001**
PD-MCI	0.48	0.6	-	**0.001**	**<0.001**
PDD	**0.031**	**0.032**	**0.035**	-	0.63
DLB	**<0.001**	**<0.001**	**<0.001**	**0.033**	-
p-tau (T+) ~ age + group					
	CTRL	PD-nMCI	PD-MCI	PDD	DLB
CTRL	-	0.42	0.72	0.06	**0.002**
PD-nMCI	0.27	-	0.94	0.09	**0.009**
PD-MCI	0.54	0.89	-	0.06	**0.003**
PDD	0.91	0.78	0.94	-	0.31
DLB	**0.004**	**0.018**	**0.016**	0.13	-
t-tau (N+) ~ age + group					
	CTRL	PD-nMCI	PD-MCI	PDD	DLB
CTRL	-	0.06	0.3	0.14	**0.0075**
PD-nMCI	0.51	-	0.22	0.71	0.42
PD-MCI	0.17	0.1	-	0.61	0.07
PDD	0.31	0.23	0.75	-	0.27
DLB	0.33	0.83	**0.033**	0.097	-
A+/T+ ~ age + group					
	CTRL	PD-nMCI	PD-MCI	PDD	DLB
CTRL	-				
PD-nMCI		-	0.47	**0.038**	**0.009**
PD-MCI			-	**0.049**	**0.01**
PDD				-	0.51
DLB					-

**Table 3:** Summary of MoCA, MMSE, UPDRS-II and H&Y scores, together with the number of patients who underwent neuropsychological and clinical assessment, at one-year follow-up (T1). Mean values at T1 and mean differences (Δ) with respect to baseline evaluations are reported for PD-nMCI and PD-MCI groups with their 95% CI.

Test at T1	PD-nMCI			PD-MCI		
	N	Mean value (95% CI)	Mean Δ (95% CI)	N	Mean value (95% CI)	Mean Δ (95% CI)
MoCA	25	25.7 (24.4, 27.0)	0.6 (−0.16, 1.36)	21	19.6 (18, 21.2)	0.57 (−0.75, 1.9)
MMSE	25	28.5 (27.9, 29.2)	0.32 (−0.26, 0.9)	21	25.9 (24.6, 27.1)	−0.14 (−1.17, 0.89)
UPDRS-III	31	24.7 (21.3, 28.1)	−2.6 (−6.2, 1.1)	29	29.2 (26.4, 32)	0.14 (−2.54, 2.82)
H&Y	31	2.1 (1.9, 2.3)	0 (−0.16, 0.16)	29	2.2 (2, 2.3)	−0.08 (−0.25, 0.08)

## Supplementary Materials

The following are available online at https://www.mdpi.com/2075-4418/10/12/1015/s1, Table S1. Description of the neuropsychological battery used to classify PD-nMCI and PD-MCI patients, Table S2. Number of samples analysed with ELISA and Lumipulse G600-II (Fujirebio Inc.) for the considered diagnostic groups, Table S3. Differences in biomarker values within the PD group between measurements performed with ELISA and Lumipulse, Figure S1. Distributions of Aβ42/Aβ40 values measured with EUROIMMUN ELISA and Lumipulse in the PD cohort, Figure S2. Distributions of p-tau values measured with INNOTEST ELISA and Lumipulse in the PD cohort, Figure S3. Distributions of t-tau values measured with INNOTEST ELISA and Lumipulse in the PD cohort, Table S4. Age-adjusted non-parametric p-values relative to biomarker differences between different diagnostic groups are represented in a matrix-like fashion.

## Abbreviations

A+	amyloidosis
AD	Alzheimer’s disease
ANCOVA	analysis of covariance
ANOVA	analysis of variance
Aβ	amyloid beta protein
CI	confidence interval
CNS	central nervous system
COMT-I	catechol-o-methyltransferase inhibitors
CSF	cerebrospinal fluid
CTRL	control subjects
DA	dopamine agonists
DLB	dementia with Lewy bodies
ELISA	enzyme-linked immunosorbent assay
H&Y	Hohen and Yahr scale
LB	Lewy bodies
L-dopa	levodopa
LEDD	levodopa Equivalent Daily Dose
LP	lumbar puncture
MAO-B-I	monoamine oxidase inhibitors
MCI	mild cognitive impairment
MMSE	mini-Mental State Examination
MoCA	Montreal Cognitive Assessment
N+	neurodegeneration
NIA-AA	National Institute on Aging—Alzheimer’s Association
PD	Parkinson’s Disease
PDD	Parkinson’s Disease with dementia
PD-MCI	Parkinson’s Disease with mild cognitive impairment
PD-nMCI	cognitively unimpaired Parkinson’s Disease
PD-T	treated PD patients
PET	positron emission tomography
p-tau	tau protein phosphorylated at threonine 181
T+	tauopathy
t-tau	total tau protein
