# Supplementary material for: A/T/(N) Profile in Cerebrospinal Fluid of Parkinson’s Disease with/without Cognitive Impairment and Dementia with Lewy Bodies
Source: Diagnostics (Basel). 2020 Nov 26;10(12):1015. doi: 10.3390/diagnostics10121015 (PMC7760640; doi:10.3390/diagnostics10121015)
Supplement: Supplementary file 1 [file diagnostics-10-01015-s001.pdf]

diagnostics

MDPI

A/T/(N) Profile in Cerebrospinal Fluid of Parkinson's Disease with/without Cognitive Impairment and Dementia with Lewy Bodies

Giovanni Bellomo [1,†], Federico Paolini Paoletti [2,†], Elena Chipi [2], Maya Petricciuolo [1], Simone Simoni [2], Nicola Tambasco [2] and Lucilla Parnetti [1,2,*]

1 Laboratory of Clinical Neurochemistry, Section of Neurology, Department of Medicine, University of Perugia, 06132, Perugia (PG), Italy
2 Section of Neurology, Department of Medicine, University of Perugia, 06132, Perugia (PG), Italy
* Correspondence: lucilla.parnetti@unipg.it
† These authors equally contributed

Table 1. Description of the neuropsychological battery used to classify PD-nMCI and PD-MCI patients. The battery used is comprehensive of two tests within each of the five domains (attention and working memory, executive, language, memory and visuo-spatial), according to the Movement Disorder Society (MDS) recommendations criteria.

Cognitive Domains	Neuropsychological Tests
Attention and working memory	Trail Making Test [1]
	Digit Span forward/backward [2]
Executive functions	Verbal fluency test [3]
	Stocking of Cambridge from Cambridge Neuropsychological Test Automated Battery (CANTAB) [4–6]
Language	WAIS-IV Similarities [7]
	Semantic fluency [3]
Memory	Rey's Auditory Verbal Learning [8]
	Prose recall test [3]
Visuo-spatial	Clock copying [9]
	Copying drawings/Copying drawing with landmarks from The Mental Deterioration Battery (MDB) [10]

Table 2. Number of samples analysed with enzyme-linked immunoassay (ELISA) and Lumipulse G600-II (Fujirebio Inc.) for the considered diagnostic groups. No significant differences, in terms of fractions of samples measured with ELISA/Lumipulse, were found for each biomarker between groups pairs by Fisher's exact test for count data [11]. Acronyms: Parkinson's disease (PD), phosphorylated tau (p-tau), total tau (t-tau), amyloid-β (Aβ), controls (CTRL), cognitively unimpaired Parkinson's disease patients (PD-nMCI), Parkinson 'disease patients with mild cognitive impairment (PD-MCI), Parkinson's disease with dementia (PDD), dementia with Lewy bodies (DLB).

Group	Aβ42/Aβ40 Lumipulse/ELISA	p-tau Lumipulse/ELISA	t-tau Lumipulse/ELISA
CTRL	25/23	25/23	25/23
PD-nMCI	30/20	20/30	30/20
PD-MCI	32/16	16/32	32/18
PDD	7/7	7/7	7/7
DLB	7/8	7/8	7/8

Table 3. Differences in biomarker values within the PD group between measurements performed with ELISA and Lumipulse. Both parametric (Student's *t*-test) and non-parametric tests (Wilcoxon's W-test) were applied since normality could be excluded by the Kolmogorov-Smirnov (K-S) test for t-tau values. Although mean biomarker values did not significantly differ between the two assays, Lumipulse G amyloid-β (Aβ)42/Aβ40 and p-tau exhibited slightly lower values with respect to EUROIMMUN and INNOTEST ELISA, respectively.

Biomarker	ELISA PD-nMCI/PD-MCI	Lumipulse PD-nMCI/PD-MCI	ELISA Mean (95%CI)	Lumipulse Mean (95%CI)	Normality K-S	t-Test	W-Test
**Aβ42/Aβ40**	20/16	30/32	0.110 (0.10, 0.120)	0.102 (0.095, 0.107)	yes	0.1	-
**p-tau**	32/30	18/20	41.7 (36.9, 46.5) pg/mL	35.1 (29.16, 41.1) pg/mL	yes	0.08	-
**t-tau**	20/16	30/32	271 (217, 326) pg/mL	317 (218,416) pg/mL	no	-	0.86

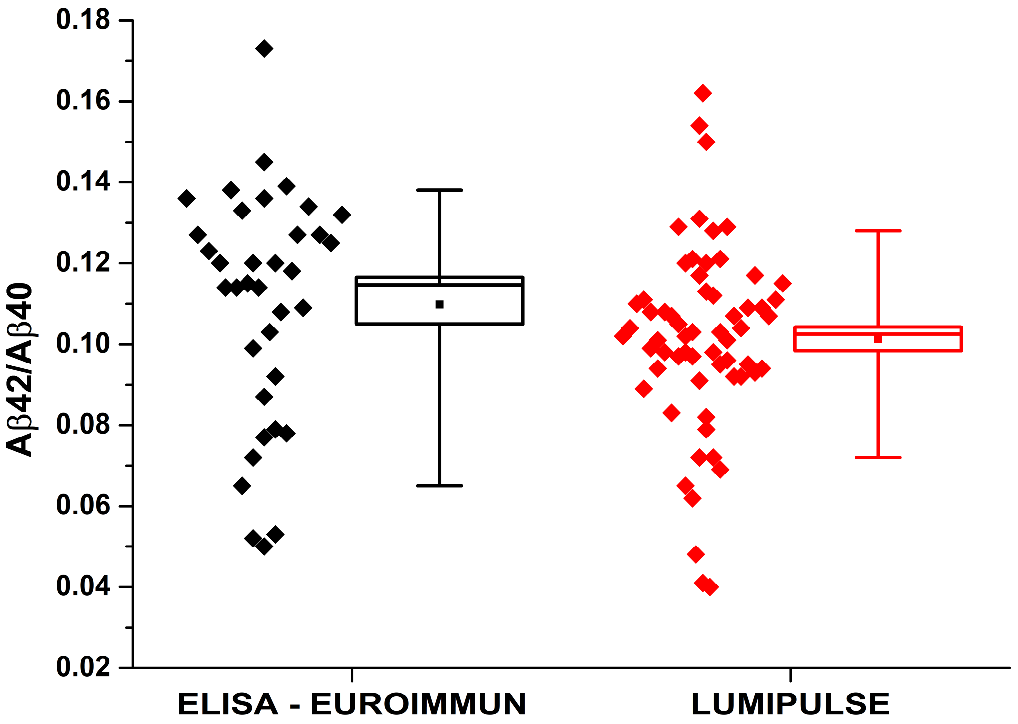

Figure 1. Distributions of Aβ42/Aβ40 values measured with EUROIMMUN ELISA and Lumipulse in the PD cohort. Boxes representing data distributions are centred on the mean values, with the internal horizontal line representing the median. Boxes heights are equal to the standard error of mean values while whiskers represent the 10–90% data range.

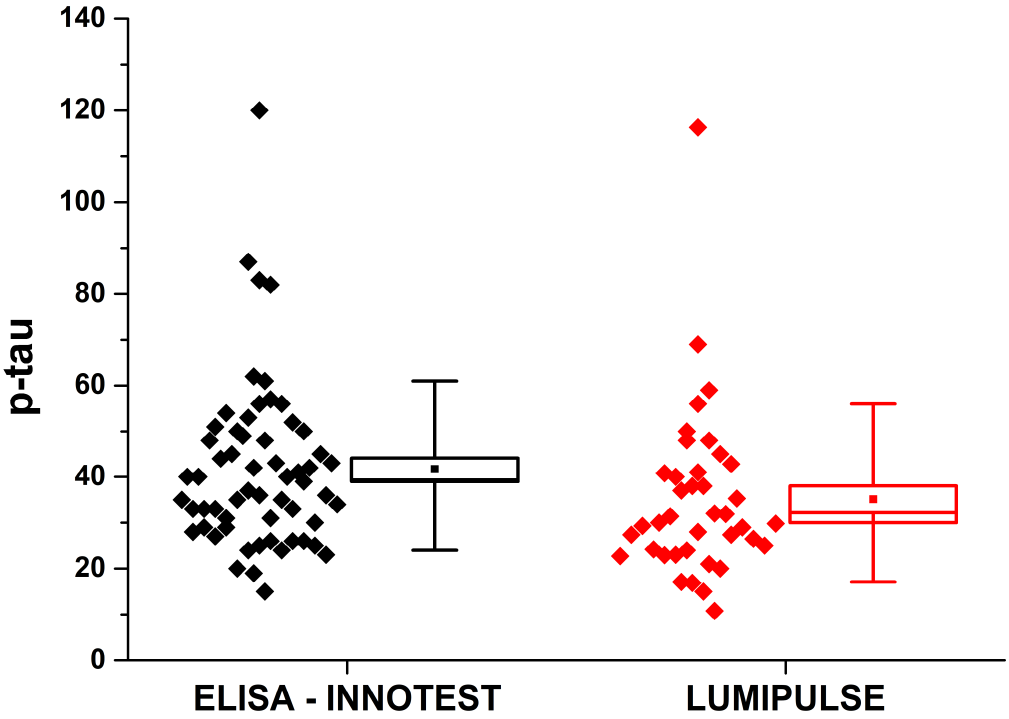

Figure 2. Distributions of phosphorylated tau (p-tau) values measured with INNOTEST ELISA and Lumipulse in the PD cohort. Boxes representing data distributions are centred on the mean values, with the internal horizontal line representing the median. Boxes heights are equal to the standard error of mean values while whiskers represent the 10–90% data range.

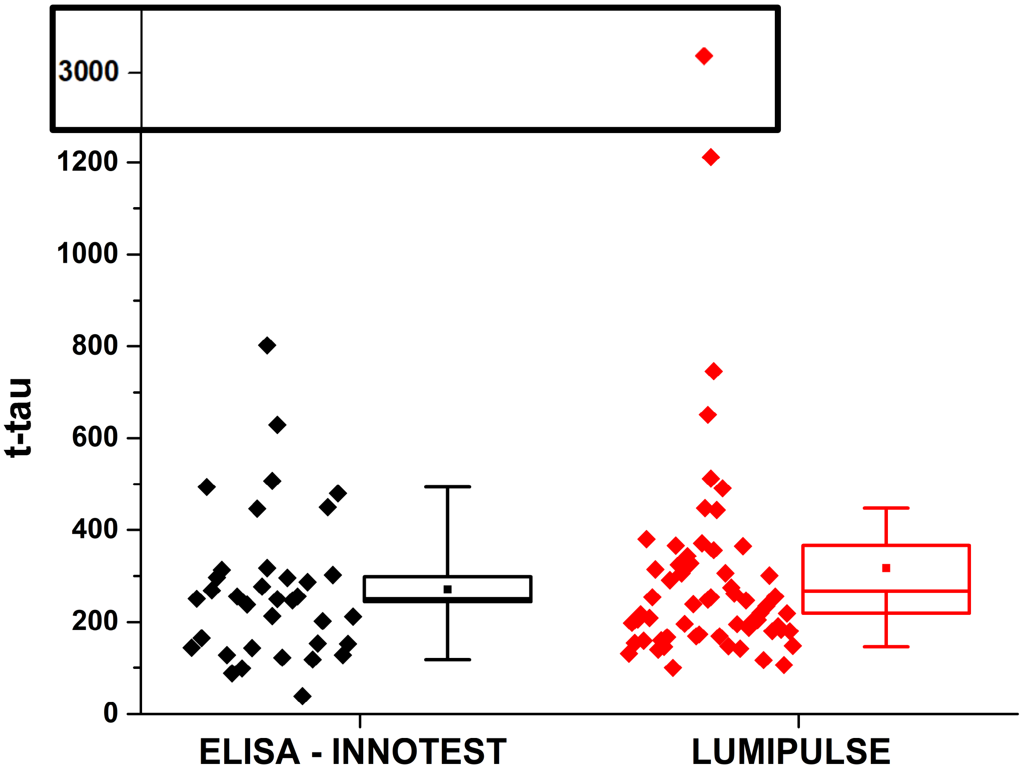

Figure 3. Distributions of t-tau values measured with INNOTEST ELISA and Lumipulse in the PD cohort. Boxes representing data distributions are centred on the mean values, with the internal horizontal line representing the median. Boxes heights are equal to the standard error of mean values while whiskers represent the 10–90% data range.

Table 4. Age-adjusted p-values relative to biomarker differences between different diagnostic groups are represented in a matrix-like fashion. The p-values inserted in the lower diagonal part of the matrices (green cells) are relative to continuous markers (Aβ42/Aβ40, p-tau and t-tau) while the p-values inserted in the upper diagonal part are relative to amyloidosis/tauopathy/(neurodegeneration) - A/T/(N) prevalences (magenta cells). Differences in continuous markers between diagnostic groups were assessed nonparametric analysis of covariance (ANCOVA) [12] by controlling for age. Differences in A/T/(N) binary outcomes between diagnostic groups were assessed by binomial logistic regression by controlling for age. Cells relative to significant p-values (<0.05) are represented in bold.

**Continuous marker**			**A/T/(N)**		
**Aβ42/Aβ40 (A+) ~ age + group**					
	CTRL	PD-nMCI	PD-MCI	PDD	DLB
CTRL	**-**	0.42	0.44	**<0.001**	**<0.001**
PD-nMCI	0.35	-	0.81	**0.003**	**0.001**
PD-MCI	0.48	0.87	-	**0.001**	**<0.001**
PDD	**0.016**	0.1	**0.019**	-	0.63
DLB	**0.002**	**0.003**	**0.011**	0.1	
**p-tau (T+) ~ age + group**					
	CTRL	PD-nMCI	PD-MCI	PDD	DLB
CTRL	-	0.42	0.72	0.06	**0.002**
PD-nMCI	0.15	-	0.94	0.09	**0.009**
PD-MCI	0.34	0.52	-	0.06	**0.003**
PDD	0.28	0.94	0.54	-	0.31
DLB	**0.005**	**0.0049**	**0.0054**	0.91	
**t-tau (N+) ~ age + group**					
	CTRL	PD-nMCI	PD-MCI	PDD	DLB
CTRL	-	0.06	0.3	0.14	**0.0075**
PD-nMCI	0.27	-	0.22	0.71	0.42
PD-MCI	0.46	0.09	-	0.61	0.07
PDD	0.59	0.62	0.95	-	0.27
DLB	0.41	0.23	**0.01**	0.88	
**A+/T+ ~ age + group**					
	CTRL	PD-nMCI	PD-MCI	PDD	DLB
CTRL	-				
PD-nMCI		-	0.47	**0.038**	**0.009**
PD-MCI			-	**0.049**	**0.01**
PDD				-	0.51
DLB					

References

1. Giovagnoli, A.R.; Del Pesce, M.; Mascheroni, S.; Simoncelli, M.; Laiacona, M.; Capitani, E. Trail making test: normative values from 287 normal adult controls. *Ital J Neurol Sci* **1996**, *17*, 305–309, doi:10.1007/BF01997792.
2. Monaco, M.; Costa, A.; Caltagirone, C.; Carlesimo, G.A. Forward and backward span for verbal and visuo-spatial data: standardization and normative data from an Italian adult population. *Neurol Sci* **2013**, *34*, 749–754, doi:10.1007/s10072-012-1130-x.
3. Novelli, G.; Papagno, C.; Capitani, E.; Laiacona, M.; et al Tre test clinici di ricerca e produzione lessicale. Taratura su sogetti normali. [Three clinical tests to research and rate the lexical performance of normal subjects.]. *Archivio di Psicologia, Neurologia e Psichiatria* **1986**, *47*, 477–506.

4. Morris, R.G.; Evenden, J.L.; Sahakian, B.J.; Robbins, T.W. Computer-aided assessment of dementia: Comparative studies of neuropsychological deficits in Alzheimer-type dementia and Parkinson's disease. In *Cognitive neurochemistry*; Oxford University Press: New York, NY, US, 1987; pp. 21–36 ISBN 978-0-19-854225-4.
5. Sahakian, B.J.; Morris, R.G.; Evenden, J.L.; Heald, A.; Levy, R.; Philpot, M.; Robbins, T.W. A comparative study of visuospatial memory and learning in Alzheimer-type dementia and Parkinson's disease. *Brain* **1988**, *111 (Pt 3)*, 695–718, doi:10.1093/brain/111.3.695.
6. CANTAB Cognitive Research Software Available online: https://www.cambridgecognition.com/cantab/ (accessed on Nov 2, 2020).
7. Petermann, F. *Wechsler Adult Intelligence Scale - fourth edition (WAIS-IV): Manual zur Durchführung und Auswertung ; deutsche Übersetzung und Adaptation der WAIS-IV von David Wechsler ; [PsychCorp]*; Pearson, 2012;
8. Carlesimo, G.A.; Sabbadini, M.; Fadda, L.; Caltagirone, C. Different Components in Word-List Forgetting of Pure Amnesics, Degenerative Demented and Healthy Subjects. *Cortex* **1995**, *31*, 735–745, doi:10.1016/S0010-9452(13)80024-X.
9. Caffarra, P.; Gardini, S.; Zonato, F.; Concari, L.; Dieci, F.; Copelli, S.; Freedman, M.; Stracciari, A.; Venneri, A. Italian norms for the Freedman version of the Clock Drawing Test. *J Clin Exp Neuropsychol* **2011**, *33*, 982–988, doi:10.1080/13803395.2011.589373.
10. Carlesimo, G.A.; Caltagirone, C.; Gainotti, G.; Fadda, L.; Gallassi, R.; Lorusso, S.; Marfia, G.; Marra, C.; Nocentini, U.; Parnetti, L. The Mental Deterioration Battery: Normative Data, Diagnostic Reliability and Qualitative Analyses of Cognitive Impairment. *ENE* **1996**, *36*, 378–384, doi:10.1159/000117297.
11. Mehta, C.R.; Patel, N.R. A Network Algorithm for Performing Fisher's Exact Test in r × c Contingency Tables. *Journal of the American Statistical Association* **1983**, *78*, 427–434, doi:10.2307/2288652.
12. Young, S.G.; Bowman, A.W. Non-Parametric Analysis of Covariance. *Biometrics* **1995**, *51*, 920–931, doi:10.2307/2532993.
